# An update of the managment of hypermobility in children

**DOI:** 10.1186/1546-0096-12-S1-I25

**Published:** 2014-09-17

**Authors:** Sue Maillard

**Affiliations:** 1 Paediatric Rheumatology, Great Ormond Street Hospital for Children Foundation Trust, London, UK

## 

The management of symptoms potentially related to hypermobility frequently require a Biopsychosocial model of management. This intervention may be provided by a variety of professionals depending upon the individual service provision; however the principals encourage an approach that promotes independent self-management.

It is becoming increasingly understood that the degree of flexibility is not as important in predicting symptoms and outcomes as the degree of muscle strength and stamina as well as psychosocial factors such as levels of anxiety and low mood. It is not clear how many children with hypermobility are affected by symptoms, however musculoskeletal pain is common in young people.

The principals of the physical treatments should be based around ensuring correct biomechanics are maintained with individual strengthening programmes which are then supported by paced integration into sport and physical activity. Fulltime School should be the goal for all young people with hypermobility as well as inclusion into most activities. The use of aids and adaptations including wheelchairs and crutches should be avoided as this actually promotes muscle weakness and a long term increase in symptoms. It is important that a good sleeping pattern should be restored and then maintained and the use of Active Relaxation Techniques are very effective.

Teaching the young person and their family about pain and its non-pharmaceutical management is very helpful in empowering them to manage independently and not to fear pain but to be in control and therefore not limited by it.

Hypermobility should be a condition that is self-managed by the young person and their family but the professionals are extremely important in ensuring this approach is understood and effective and it is their responsibility to ensure unnecessary drugs, surgeries or treatments are given to each child. The majority of young people with symptoms related to their hypermobility respond extremely well with this approach.

## Disclosure of interest

None declared.

